# Climate worry: associations with functional impairment, pro-environmental behaviors and perceived need for support

**DOI:** 10.1186/s40359-024-02244-0

**Published:** 2024-12-09

**Authors:** Fabian Lenhard, Lorena Fernández de la Cruz, Tove Wahlund, Erik Andersson, Johan Åhlén, Francesco Fuso Nerini, Haluk Akay, David Mataix-Cols

**Affiliations:** 1 Centre for Psychiatry Research, Department of Clinical Neuroscience, Karolinska Institutet, & Stockholm Health Care Services, Region Stockholm, Sweden; 2 Division of Psychology, Department of Clinical Neuroscience, Karolinska Institutet, Stockholm, Sweden; 3 Department of Global Public Health, Karolinska Institutet, Stockholm, Sweden; 4 Center for Epidemiology and Community Medicine, Stockholm Health Care Services, Region Stockholm, Sweden; 5 KTH Climate Action Centre & KTH Division of Energy Systems, KTH Royal Institute of Technology, Stockholm, Sweden; 6 Department of Clinical Neuroscience, Karolinska Institutet, Stockholm, Sweden; 7 Environmental Change Institute, University of Oxford, Oxford, UK

**Keywords:** Climate change, Climate worry, Climate anxiety, Pro-environmental behaviors, Impairment, Need for support, Intervention

## Abstract

**Background:**

A large proportion of individuals experience functional impairment in everyday life due to climate worry. However, the current understanding of this functional impairment is limited by the use of suboptimal measures. Furthermore, it is not known whether functional impairment due to climate worry affects pro-environmental behaviors (PEBs) or whether individuals who experience such impairment perceive a need for support. The aims of the current study were (1) to extend previous research using an established measure of functional impairment (the Work and Social Adjustment Scale, WSAS), (2) to explore the associations between climate worry, functional impairment, and PEBs, and (3) to describe the characteristics and the perceived need for support of individuals with functional impairment due to climate worry.

**Methods:**

A cross-sectional survey targeting adult individuals who experience climate worry. Participants were recruited nationally in Sweden between September and October 2022. The survey included measures of climate worry severity, climate worry frequency, functional impairment, PEBs, depressive symptoms, sleep problems, and questions related to perceived need for support.

**Results:**

A total of 1221 adults (75% women, mean age 46.3 years) were included in the analyses. Multivariate structural equation modeling revealed that climate worry severity and frequency were significantly associated with PEBs (β = 0.34 and β = 0.45, respectively). Climate worry frequency was associated with functional impairment (β = 0.41). Functional impairment was only marginally associated with PEBs (β = 0.05). Approximately 40% of the sample (*n* = 484) reported a high frequency and high severity of climate worry. Among these, one-third (*n* = 153) scored above the cutoff for significant impairment on the WSAS. Individuals in this group (high severity and frequency of climate worry as well as significant functional impairment) were more likely to experience depressed mood and sleep problems and were more interested in receiving support, specifically concerning strategies for worry management and sustainable behavior change.

**Conclusions:**

Using an established measure of functional impairment, we found an association of climate worry with functional impairment and PEBs. Importantly, as there is a perceived need for support in individuals with impairment due to climate worry, interventions targeting this specific subgroup should be developed.

**Supplementary Information:**

The online version contains supplementary material available at 10.1186/s40359-024-02244-0.

## Highlights

We conducted a national survey in Sweden to study the associations between climate worry, functional impairment, and pro-environmental behaviors (PEBs).

We also investigated the characteristics and perceived need for support in the subgroup of individuals who experienced everyday life functional impairments due to frequent and severe climate worry.

We found that climate worry was significantly and positively correlated with impairment and PEBs. However, impairment did not correlate with PEBs.

We confirmed that individuals who experience functional impairment due to frequent and severe climate worry express a need for support, specifically concerning strategies for worry management and sustainable behavior strategies.

These findings are important for the development of suitable supportive interventions for this population.

## Introduction

The changing climate increasingly affects human health and wellbeing through the disruption of ecological, geographical, and societal systems [1]. While there is an extensive body of research on the climate’s impact on physical health, there is a growing demand to understand and address its mental health challenges [2].

Worry is one of several psychological reactions to climate change and its current and projected consequences. The terms “climate anxiety” and “climate worry” are often used interchangeably. However, consistent with recent suggestions to adhere to established psychological terminology [3], climate anxiety is a broader concept that includes emotional, cognitive, physiological, and behavioral components, whereas worry is defined as a cognitive process concerning something impending or anticipated [3, 4].

In the context of climate change, climate worry is not considered a pathological condition (i.e., not a clinical diagnosis) but rather an adequate reaction to a real and salient threat [5]. The prevalence of climate worry has increased significantly during the last decade, likely because of the changing climate itself and increased media coverage [6, 7]. International surveys show that, in the majority of countries, most people can now be categorized as “alarmed” or “concerned” about the climate [8]. Women and younger individuals tend to experience higher levels of climate worry [9, 10]. Geographically, respondents from South America, southern Europe, and southern Asia currently exhibit the highest levels of worry [8]. In Sweden, a representative annual survey from 2012 showed that 39% of adults felt “very worried” about climate change. By 2023, that number had increased to 51% [11].

The available data from North America, Europe, and Africa suggest that a significant proportion of the population (17–27%) experiences everyday life impairments associated with persistent climate worry, including impairments in family life, social relationships, and reduced ability to work [9, 12]. A global representative survey in ten countries including 10,000 young adults (16 to 25 years of age) revealed that almost 60% felt “very” or “extremely” worried about climate change, and 45% said that their feelings about climate change negatively affected their daily functioning [10].

However, the impact of climate worry on everyday life functioning has not been sufficiently studied using established and psychometrically sound measures. In the context of large-scale surveys, functional impairment has often been assessed using single-item measures (e.g., *“My feelings about climate change negatively affect my daily life”*) with a yes/no answer format [10]. While these measures are useful for large-scale data collection, they lack psychometric validation and comparability. Another measure that has been used to assess functional impairment is the dimensional Climate Anxiety Scale (CAS, [12]). However, several important methodological weaknesses of the CAS have been noted, primarily regarding uncertainties in its factor structure [13–15] and varying cross-cultural performance [13]. Additionally, cutoff scores that indicate significant functional impairment are currently unavailable for the CAS. Generally, impairment measures targeted specifically toward climate worry are by design limited to this specific population and do not allow for comparisons between different populations and contexts. Thus, using a well-known and psychometrically validated measure to further explore functional impairment related to climate worry would add value to the field.

Furthermore, it is well established that there is a small to moderate association between climate worry and pro-environmental behaviors (PEBs). This association has been shown cross-culturally [16] and in longitudinal studies [17]. However, little is known about whether functional impairment related to climate worry interferes with individual-level PEBs.

The aim of the current study was therefore to (1) extend previous research on the functional impact of climate worry using an established, validated measure, (2) explore associations between climate worry, functional impairment, and PEBs, and (3) describe the characteristics and the perceived need for support among individuals with functional impairment due to severe and frequent climate worry.

## Materials and methods

### Study design

We conducted an open online survey in Sweden. The survey, entitled “Are you worried about the climate?”, targeted individuals who experienced worry related to climate change. Advertisements for study participation were posted on social media (Facebook and Instagram) for two 3-day periods in September and October 2022.

### Survey content

The survey presented a series of questions to measure the relevant variables, as listed below, followed by a section collecting the gender and age of the respondents, as well as questions about their exposure to extreme weather events (“Have you come into direct contact with extreme weather events that can be assumed to be caused by climate change (e.g., floods, heat waves, forest fires, extreme storms)?”, response categories “yes/no”) and their beliefs about the role of humans in climate change (“What is your opinion about the following statement? “Climate change is mainly caused by human activities.“, response categories “true”, “somewhat true”, “false”). An English translation of the full survey is available as supplementary material.

For comparability with previous studies [9–11], we used single-item questions to measure *severity of climate worry* (“How worried do you feel about climate change and its consequences?”, response categories “very worried”, “moderately worried”, “slightly worried”, “not at all worried”), *climate worry frequency* (“How often do you worry about the climate?”, response categories “every day”, “several times a week”, “several times a month”, “rarely”, “never”), and the *perceived impact of climate worry on everyday life* (“My feelings about climate change negatively affect my daily life”, response categories “yes” and “no”).

*Functional impairment* was measured using an adapted version of the Working and Social Adjustment Scale (WSAS) [18]. The WSAS was chosen because it is an established measure of functional impairment that has been used in a broad range of behavioral and psychological studies. It is a short, generic measure of impairment and has been validated for adults [18] as well as children and adolescents [19]. The WSAS was adapted to climate worry according to the following instruction: “Below are questions regarding different everyday situations. Think carefully about each question and mark how much your everyday life is affected by climate worry.” The five items of the WSAS correspond to five domains of impairment in everyday life: work or studies, household management, social leisure activities, private leisure activities, and relationships. Items are scored from 0 to 8 on a Likert scale, with higher scores indicating more severe impairment and a maximum total score of 40. A total score ≥ 10 has been defined as indicative of functional impairment [18]. In our sample, the internal consistency of the WSAS was Cronbach’s alpha = 0.91. As this was the first time the WSAS was used in this specific context, we conducted a confirmatory factor analysis, confirming the one factor structure of the scale (see supplementary material).

Adapted from previously established methodology [20], *PEBs* were measured using five questions covering a range of different domains, including household-related behaviors (e.g., recycling, saving electricity), consumption-related behaviors (e.g., buying ecological products, choosing a plant-based diet), transportation (e.g., taking the train instead of flying), public sphere actions (e.g., demonstrating, signing a petition), and organizational-level actions (e.g., being involved in a voluntary organization). Each item was rated on a 4-point Likert scale to indicate the occurrence of PEBs during the past two weeks, from 0 (not at all) to 3 (often). A Cronbach’s alpha = 0.83 demonstrated good internal consistency. A factor analysis of the five items indicated one underlying factor (see supplementary material). We therefore proceeded to combine the five PEB items into one composite score.

*Depressed mood* was measured using the Patient Health Questionnaire 2-item version (PHQ-2) [21], a short version of the PHQ-9 [22]. The PHQ-2 consists of two questions associated with core symptoms of depression (“1. Little interest or pleasure in doing things.” and “2. Feeling down, depressed, or hopeless.”), scored on a 5-point Likert scale (“not at all”, “several days”, “more than half of the days”, “nearly every day”). The recommended total score cutoff of ≥ 2 was used to screen for individuals with clinical levels of depression [21]. The internal consistency in our sample was Cronbach’s alpha = 0.75.

*Sleep problems* were measured by the Insomnia Sleep Index - Short version (ISI-S) [23]. The ISI-S consists of two items concerning satisfaction/dissatisfaction with one’s current sleep pattern and interference of sleep problems with daily functioning (Item 1: “How satisfied/dissatisfied are you with your current sleep pattern?”, response categories “very satisfied”, “satisfied”, “moderately satisfied”, “dissatisfied”, “very dissatisfied”; and item 2: “To what extent do you consider your sleep problem to interfere with your daily functioning (e.g. daytime fatigue, mood, ability to function at work/daily chores, concentration, memory, mood, etc.) currently?”, response categories “not at all interfering”, “a little”, “somewhat”, “much”, “very much interfering”). It correlates highly with the full version of the scale, and a total score cutoff of ≥ 6 has demonstrated good specificity and sensitivity when used as a screening measure for insomnia [23]. The internal consistency in our sample was Cronbach’s alpha = 0.82.

*Perceived need for support.* Participants were asked whether they had been in contact with healthcare services regarding problems with climate worry (“Have you been in contact with healthcare services to ask for help for your worry related to climate change?”, response categories “yes”, “no”) and their interest in strategies for the management of climate worry and sustainability strategies (“How interested are you in the following topics? Climate change, its causes and consequences; Climate psychology: Thought traps and psychological challenges of climate change; Strategies for coping with climate worry more constructively; How you as an individual can develop a more sustainable lifestyle; What you can do together with others to build a more sustainable community; How to talk to children about climate change.”, response categories “not interested at all”, “a little interested”, “very interested”).

### Participants

Participants included in the study sample were individuals aged 18 years or older who provided informed consent to participate and completed the entire survey. Participant data were collected anonymously. The survey system did not allow several answers from the same IP address to avoid multiple answers from the same individual or answers from internet bots or algorithms. Additionally, the survey included an “I am not a robot” challenge (an image with a simple mathematical question, “5 + 1 =?”) that all respondents had to answer to proceed to prevent automated responses.

A total of 1,366 individuals started the survey, and 1,229 answered all questions and were therefore included in the analyses (90% response rate). After eliminating respondents outside the age range (i.e., < 18), the full study sample included 1,221 respondents with a mean age of 46.3 years (SD = 13.1, range 18–82). A total of 915 (75%) were women, 284 (23%) were men, and 22 (1.8%) identified as nonbinary/nonconforming. Table 1 shows the sample characteristics on the assessment measures.

**Table 1:** Sample characteristics on the assessment measures

Measure	Response categories	M (SD)/Percentage	*n*
Climate worry frequency	every day	42.8%	523
	several times a week	33.7%	412
	several times a month	7.0%	86
	rarely	5.1%	62
	never	11.3%	138
Climate worry severity	very worried	64.0%	781
	moderately worried	19.3%	236
	slightly worried	5.7%	70
	not at all worried	11.0%	134
WSAS	Total score	4.73 (6.16)	1,221
PEBs	Total score	10.12 (4.16)	1,221
PHQ-2	Total score	1.33 (1.50)	1,221
ISI-S	Total score	3.20 (1.99)	1,221
Perceived impact of climate worry on everyday life	yes	46.2%	564
	no	53.8%	657
Perception of being exposed to extreme, climate change-related weather events	yes	45.9%	560
	no	54.1%	661
Belief that climate change is human-caused	true	80.7%	985
	somewhat true	8.7%	106
	false	10.6%	130

*Abbreviations*: WSAS = Work and Social Adjustment Scale, PEBs = Pro-Environmental Behaviors, PHQ-2 = Patient Health Questionnaire 2-item version, ISI-S = Insomnia Severity Index – Short version

### Data analysis

Descriptive statistics were analyzed as frequencies and percentages or means and standard deviations, as appropriate. Cronbach’s alpha was used as an estimate of internal consistency of the dimensional measures. To extend previous research on the functional impact of climate worry using an established, validated measure (aim 1), we used Spearman correlations between a previously used single-item question of functional impairment and the WSAS. Significance levels were Bonferroni-adjusted. Associations between climate worry (severity and frequency), functional impairment, and PEBs (aim 2) were analyzed using structural equation modeling (SEM) with the maximum likelihood method, and standard errors were estimated with the observed information matrix (OIM) algorithm. To describe the characteristics of individuals who experienced functional impairment due to severe and frequent climate worry (aim 3), we separated the sample into a subgroup who reported that they worried “very much” and “every day” about climate change and scored above the cutoff for significant functional impairment on the WSAS. We compared this subgroup to the remaining sample regarding age, sex, exposure to extreme weather events, beliefs about the role of humans in climate change, depressed mood (PHQ-2) and sleep problems (ISI-S). To describe the perceived need for support among the individuals who experienced functional impairment due to climate worry (aim 3), we compared the reported interest in worry management and sustainability strategies between those who reported frequent and persistent climate worry and functional impairment with the remaining sample. Associations between categorical variables were analyzed using Fischer´s exact test or linear regression if the outcome was continuous. The analyses were carried out in Stata, version 15 [24] and R [25].

## Results

### Intercorrelations between study constructs (aim 1)

Spearman intercorrelation coefficients between the study constructs are presented in Table 2. Strong correlations were found between climate worry severity and climate worry frequency, between climate worry severity and PEBs, between climate worry frequency and PEBs, and between the WSAS and the corresponding single-item measure of perceived impairment.

**Table 2:** Spearman correlations between study constructs

	1.	2.	3.	4.	5.	6.	7.
1. Climate worry severity	1						
2. Climate worry frequency	0.73*	1					
3. Perceived impairment (single-item measure)	0.46*	0.51*	1				
4. WSAS	0.47*	0.51*	0.65*	1			
5. PEBs	0.63*	0.65*	0.41*	0.44*	1		
6. PHQ-2	0.44*	0.48*	0.56*	0.63*	0.40*	1	
7. ISI-S	0.25*	0.29*	0.27*	0.34*	0.21*	0.34*	1

Note: * = *p* < .001, Bonferroni-adjusted significance level. Abbreviations: WSAS = Work and Social Adjustment Scale, PEBs = Pro-Environmental Behaviors

### Multivariate associations between climate worry, functional impairment, and PEBs (aim 2)

**Fig. 1:**
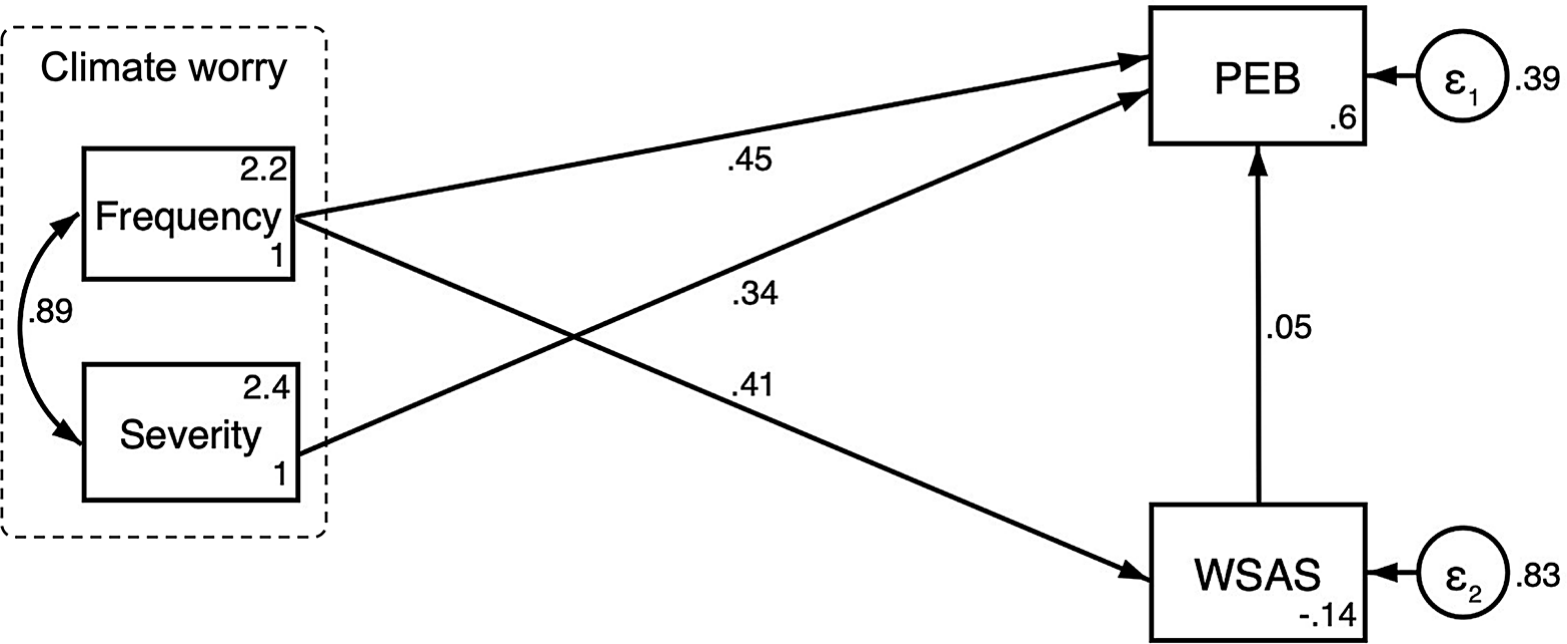
SEM analysis results (Abbreviations: PEB = Pro-Environmental Behaviors, WSAS = Work and Social Adjustment Scale)

We initially tested the saturated SEM, including all possible paths between climate worry severity, climate worry frequency, WSAS and PEBs. In this model, the path between climate worry severity and the WSAS was non-significant (see Supplemental Materials). We therefore excluded this path from the final model (see Fig. 1). The final SEM demonstrated substantial and significant paths between climate worry severity and climate worry frequency with PEBs, respectively (*p* < .001). Climate worry frequency was associated with the WSAS (*p* < .001). The WSAS was positively associated with PEBs (*p* = .011), although the magnitude of this association was negligible.

This model showed good fit to the data (χ2(1) = 0.223; *p* = .64; CFI = 1.00; TLI = 1.00; RMSEA < 0.001; SRMR = 0.002). The complete model results are presented in the Supplementary Materials.

### Characterization of individuals with functional impairment due to frequent and severe climate worry (aim 3)

To further explore the association between climate worry and functional impairment, we selected a subgroup of individuals who reported high frequency of this worry (defined as endorsing worry about the climate “every day” in the climate worry frequency question) and high severity of climate worry (defined as endorsing that they felt “very worried” about the climate in the climate worry severity question). A total of 484 (40%) individuals fulfilled these criteria. Within this group, 153 (32%) participants scored above the cutoff on the WSAS, indicating significant functional impairment.

There were significantly more women in this subgroup (high climate worry frequency, high severity, and functional impairment) than in the remaining sample (83% vs. 74%, respectively, *p* < .001). There was no significant difference in age between the two groups (*t* = -1.76, *p* = .08). On average, individuals in this subgroup were more likely to score above the cutoff on the PHQ-2 than were those in the remaining sample (85.0% vs. 29.2%, *p* < .001), and they were more likely to score above the cutoff on the ISI-S (30.1% vs. 10.2%, *p* < .001). Individuals in the subgroup endorsed more frequently that they had experienced extreme, climate change-related weather events compared to the remaining sample (63.8% vs. 43.5%, *p* < .001) and stated more often that they found human-caused climate change to be true (98.7% vs. 78.1%, *p* < .001).

**Fig. 2:**
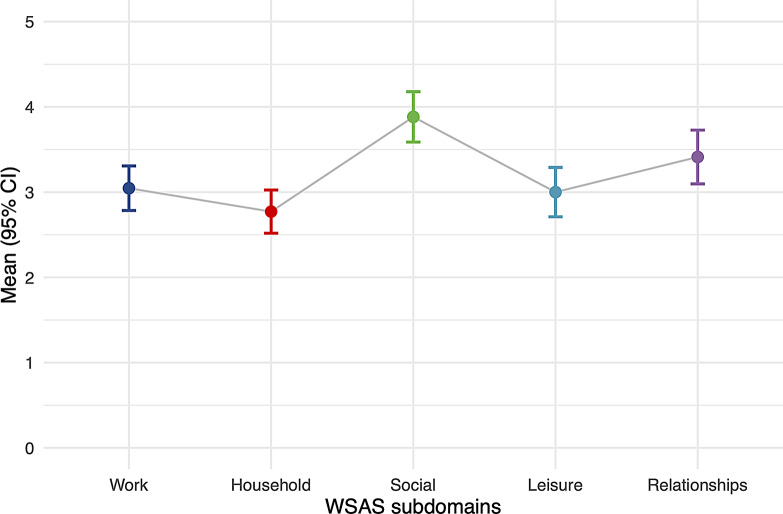
Mean item-level scores and 95% confidence intervals on the WSAS for individuals in the subgroup of individuals who experienced impairment due to frequent and severe climate worry

Figure 2 displays item-level mean scores on the WSAS for individuals in the high climate worry frequency, high climate worry severity and significant impairment subgroup. Pairwise contrasts between the five items showed that the mean value of item 3 “Social” was significantly higher than that of the remaining 4 items (*p* < .05). The difference between items 2 “Household” and 5 “Relationships” was also statistically significant (t = 3.73, *p* = .002).

### Perceived need for support among individuals with functional impairment due to frequent and severe climate worry (aim 3)

A total of 38 (25%) of those in the subgroup with high climate worry frequency, high climate worry severity and significant impairment reported that they had been in contact with healthcare services for their worry related to climate change (compared with 24 [2%] in the remaining sample, *p* < .001). A total of 118 individuals (77%) in this subgroup reported that they would be interested in learning more about strategies to manage climate worry and sustainable behaviors (compared with 538 [50%] in the remaining sample, *p* < .001). Specifically, 131 individuals (86%) in the subgroup had high interest in learning more about climate change, its causes, and consequences, 107 (70%) had high interest in the psychological aspects of climate change, 110 (72%) had high interest in strategies for the management of climate worry, 113 (74%) had high interest in individual-level sustainability, 138 (90%) had high interest in systemic-level sustainability, and 103 (67%) had high interest in how to talk with children about climate change. Interest in the above topics was significantly lower in the remaining sample (*p* < .001 for all comparisons).

## Discussion

In this study, we explored functional impairment related to climate worry using a national online survey format. The first aim was to extend previous results on functional impairment associated with climate worry using a validated measure of functional impairment. We found that the WSAS was strongly associated with single-item measures of climate worry severity, climate worry frequency and, most strongly, functional impairment.

In the multivariate structural model, we found that climate worry frequency, but not climate worry severity, was associated with the WSAS, indicating that the temporal persistence of this worry, rather than the degree of worry, is important for everyday life impairments. This appears logical, as a proven strategy to handle excessive worrying in psychological interventions is, for example, to limit worrying to a scheduled time each day [26, 27], thus temporally constraining its interference with functional cognitive and behavioral processes.

In line with previous research, we found a moderately strong association between climate worry frequency and severity with PEBs [16]. Interestingly, in the structural model, the association between functional impairment and PEBs was negligible, indicating that when interaction effects are taken into account, everyday life impairments due to climate worry may not hinder effective individual-level actions targeting climate change.

One-third of those who expressed severe and frequent climate worry scored above the cutoff for functional impairment on the WSAS. This is roughly within the range that previous studies have indicated [9, 12]. However, we could not confirm the previously suggested association between younger age and impairment [10]. Impairment due to frequent and severe climate worry was however, associated with a greater probability of depressed mood and sleep problems. This finding is in line with previous results [28, 29]; however, as has been pointed out [29], longitudinal studies are needed to clarify the causal relationship between climate worry and mental health problems. Importantly, when developing and offering interventions that target individuals with frequent and severe climate worry, cooccurring mental health needs should be taken into account.

Separate analyses of the WSAS subdomains revealed that impairment in the group with high worry and high impairment was most pronounced in the social domain. Interestingly, the study by Hickman et al. [10] reported that approximately 80% of young adults in their international sample talked with others about the climate, and almost half (48%) reported that other people had ignored or dismissed them when doing so, suggesting that the social aspects of climate change could be especially challenging to handle. An important next step would be to explore the subdomains of functional impairment further, for example, by combining high-resolution quantitative methods and qualitative interview methods.

When exploring the perceived need for support among those with frequent and severe climate worry and functional impairment, we found that one-fourth of the participants in this group had been in contact with healthcare services to receive help for their climate worry. This underlines further a previously made notion, that individuals from this group indeed seek help within regular healthcare services, and thus healthcare providers should be prepared for this relatively new demand [5]. In addition, a large majority of participants from this group expressed interest in tools for worry management and strategies for sustainable behaviors. Thus, our data clearly suggest that there is a demand for support in this group. Importantly, there is preliminary evidence that individuals can effectively learn to manage their climate worry when provided with appropriate psychological interventions [27].

## Limitations

Several limitations apply to the study methodology and results. First, our study was conducted in Sweden and may therefore be limited to similar cultural contexts and countries. Additionally, as is common in this type of sampling method, there was a large percentage of women. Hence, responses for other genders may be underrepresented. Second, we used a survey method specifically aimed at individuals feeling worried about the climate. Our results are therefore not representative of the whole population. Third, our survey was anonymous, and the identities of the respondents could not be verified. This method may present an advantage for respondents to answer freely and reduce socially desirable responses, but it could also introduce an unknown degree of random answers or response bias. Fourth, several of our study constructs were measured using single items with categorical responses, thus potentially limiting variance. Finally, our method is cross-sectional and does not allow for causal inference. An important next step would therefore be to longitudinally explore the characteristics of this population regarding vulnerability and resilience factors.

## Conclusions

Using a national survey method, our study extended previous findings on climate worry using an established measure of functional impairment. Climate worry was associated with PEBs and impairment. However, the association between impairment and PEBs was negligible. We found that a relatively large proportion of individuals with severe and frequent climate worry experienced significant impairments in everyday life functioning. The majority of individuals in this group expressed a need for support. Based on our findings, such support should involve strategies aimed toward worry management and well-being, as well as climate change knowledge and tools for sustainable behaviors. The design of supportive interventions targeted towards this group should therefore include psychological as well as sustainability components.

## Electronic supplementary material

Below is the link to the electronic supplementary material.

Supplementary Material 1

## Data Availability

The data of this study can be obtained upon reasonable request to the corresponding author.
