# Supplementary material for: Climate worry: associations with functional impairment, pro-environmental behaviors and perceived need for support
Source: BMC Psychol. 2024 Dec 9;12:731. doi: 10.1186/s40359-024-02244-0 (PMC11626749; doi:10.1186/s40359-024-02244-0)
Supplement: Supplementary file 1 — Supplementary Material 1 [file 40359_2024_2244_MOESM1_ESM.pdf]

Supplemental Materials

Full questionnaire 2
Factor analysis of pro-environmental behavior items 5
Results saturated SEM 6
Final SEM results 8

Full questionnaire
“Are you worried about climate change?”

Variable	Question	Response options
Climate worry severity	How worried do you feel about climate change and its consequences?	• Very worried<br>• Moderately worried<br>• Slightly worried<br>• Not at all worried
Climate worry frequency	How often do you worry about the climate?	• every day<br>• several times a week<br>• several times a month<br>• rarely<br>• never
Perceived climate worry impact	My feelings about climate change negatively affect my daily life (at least one of the following areas is affected: ability to concentrate, work, education, sleep, eating, leisure time, social situations)	• yes<br>• no
Extreme weather exposure	Have you come into direct contact with extreme weather events that can be assumed to be caused by climate change (e.g., floods, heat waves, forest fires, extreme storms)?	• yes<br>• no
Belief about human-caused climate change	What is your opinion about the following statement? "Climate change is mainly caused by human activities."	• True<br>• Somewhat true<br>• False
PHQ2_1	In the last two weeks, how often have you been bothered by any of the following problems related to climate change?<br>1. Little interest or pleasure in doing things	• Not at all<br>• Several days<br>• More than half of<br>• the days<br>• Nearly every day
PHQ2_2	2. Feeling down, depressed, or hopeless	• Not at all<br>• Several days<br>• More than half of<br>• the days<br>• Nearly every day
WSAS_1	Below are questions regarding different everyday situations. Think carefully about each question and mark how much your everyday life is affected by climate worry.<br>WORK / STUDY<br>1. Because of my climate worry, my ability to work or study is impaired.	0 - not at all<br>1<br>2 - slightly<br>3<br>4 - definitely<br>5<br>6 - markedly<br>7<br>8 – very severely
WSAS_2	HOME<br>2. Because of my climate worry, my home management (cleaning,	0 - not at all<br>1<br>2 - slightly

	tidying, shopping, cooking, looking after the home or the children, paying the bills) is impaired.	3<br>4 - definitely<br>5<br>6 - markedly<br>7<br>8 – very severely
WSAS_3	SOCIAL ACTIVITIES<br>3. Because of my climate worry, my social leisure activities with other people (such as parties, going to bars, clubs, outings, visits, dating, home entertainment) are impaired.	0 - not at all<br>1<br>2 - slightly<br>3<br>4 - definitely<br>5<br>6 - markedly<br>7<br>8 – very severely
WSAS_4	LEISURE ACTIVITIES<br>4. Because of my climate worry, my private leisure activities (done alone, such as reading, gardening, collecting, sewing, walking alone) are impaired.	0 - not at all<br>1<br>2 - slightly<br>3<br>4 - definitely<br>5<br>6 - markedly<br>7<br>8 – very severely
WSAS_5	RELATIONSHIPS<br>5. Because of my climate worry, my ability to form and maintain close relationships with others, including those I live with, is impaired.	0 - not at all<br>1<br>2 - slightly<br>3<br>4 - definitely<br>5<br>6 - markedly<br>7<br>8 – very severely
ISI_S_1	How SATISFIED/DISSATISFIED are you with your CURRENT sleep pattern?	• Very Satisfied<br>• Satisfied<br>• Moderately Satisfied<br>• Dissatisfied<br>• Very Dissatisfied
ISI_S_2	To what extent do you consider your sleep problem to INTERFERE with your daily functioning (e.g. daytime fatigue, mood, ability to function at work/daily chores, concentration, memory, mood, etc.) CURRENTLY?	• Not at all Interfering<br>• A Little<br>• Somewhat<br>• Much<br>• Very Much Interfering
PEB_1	In the last two weeks, how much have you been engaging in the following behaviors?<br>I did something environmentally friendly at home (e.g. recycled garbage, saved electricity / turned off electronics, lowered the indoor temperature, or similar)	• Never<br>• Rarely<br>• Sometimes<br>• Often

PEB_2	I consumed in a sustainable manner (e.g. shopped organic products, chose plant-based food instead of meat, bought used instead of new, or similar)	• Never<br>• Rarely<br>• Sometimes<br>• Often
PEB_3	I traveled environmentally friendly (e.g. rode my bike instead of driving the car, went by train instead of plane, or similar)	• Never<br>• Rarely<br>• Sometimes<br>• Often
PEB_4	I attended a demonstration, wrote a petition, shared a post on social media, or similar	• Never<br>• Rarely<br>• Sometimes<br>• Often
PEB_5	I got involved in or donated money to a voluntary organization or climate movement (e.g. Greenpeace, WWF, Fridays For Future or similar)	• Never<br>• Rarely<br>• Sometimes<br>• Often
Healthcare contact	Have you been in contact with healthcare services to ask for help for your worry related to climate change?	• yes<br>• no
Interest in support	Would you be interested in learning more about coping strategies for climate worry and sustainable behavior change?	• yes<br>• no
Support_1	How interested are you in the following topics? Climate change, its causes and consequences.	• Not interested at all<br>• A little interested<br>• Very interested
Support_2	Climate psychology: Thought traps and psychological challenges of climate change.	• Not interested at all<br>• A little interested<br>• Very interested
Support_3	Strategies for coping with climate worry more constructively.	• Not interested at all<br>• A little interested<br>• Very interested
Support_4	How you as an individual can develop a more sustainable lifestyle.	• Not interested at all<br>• A little interested<br>• Very interested
Support_5	What you can do together with others to build a more sustainable community.	• Not interested at all<br>• A little interested<br>• Very interested
Support_6	How to talk to children about climate change.	• Not interested at all<br>• A little interested<br>• Very interested
Gender	You are a	• Woman<br>• Man<br>• Non-binary/non-conforming
Age	Your age	• Free text field <number>

Factor analysis of pro-environmental behavior items

Factor analysis/correlation		Number of obs =	1,221
Method: principal-component factors		Retained factors =	1
Rotation: (unrotated)		Number of params =	5

Factor	Eigenvalue	Difference	Proportion	Cumulative
Factor1	3.04515	2.16322	0.6090	0.6090
Factor2	0.88194	0.43407	0.1764	0.7854
Factor3	0.44786	0.12018	0.0896	0.8750
Factor4	0.32768	0.03032	0.0655	0.9405
Factor5	0.29737	.	0.0595	1.0000

LR test: independent vs. saturated: chi2(10) = 2612.29 Prob>chi2 = 0.0000

Factor loadings (pattern matrix) and unique variances

Variable	Factor1	Uniqueness
VAR10_1	0.7315	0.4648
VAR10_2	0.8508	0.2761
VAR10_3	0.8049	0.3521
VAR10_4	0.7491	0.4389
VAR10_5	0.7597	0.4228

Results confirmatory factor analysis WSAS

Table S1: Results of confirmatory factor analysis using structural equation modeling.

	Coef.	Std. Err.	z	P>z	95% CI	
**WSAS_1**						
**Functional_impairment**	0,801	0,014	57,620	0,000	0,774	0,829
**_cons**	0,640	0,031	20,370	0,000	0,578	0,702
**WSAS_2**						
**Functional_impairment**	0,799	0,017	47,320	0,000	0,766	0,833
**_cons**	0,641	0,031	20,390	0,000	0,579	0,702
**WSAS_3**						
**Functional_impairment**	0,841	0,014	60,860	0,000	0,814	0,868
**_cons**	0,739	0,032	22,880	0,000	0,675	0,802
**WSAS_4**						
**Functional_impairment**	0,785	0,014	54,770	0,000	0,757	0,813
**_cons**	0,584	0,031	18,850	0,000	0,523	0,644
**WSAS_5**						
**Functional_impairment**	0,800	0,015	51,840	0,000	0,770	0,830
**_cons**	0,663	0,032	20,980	0,000	0,601	0,725

LR test of model vs. saturated: chi2(1) = 0.03, Prob > chi2 = 0.8577

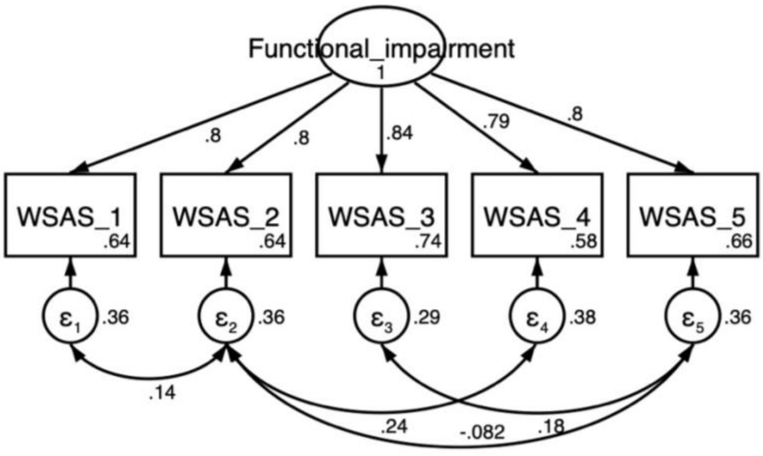

Figure S1: Confirmatory factor analysis of the WSAS.

Results saturated SEM

Table S2: SEM estimates for the saturated model

		OIM				
Standardized	Coef.	Std. Err.	z	P>z	[95% Conf.	Interval]
Structural						
WSAS_total						
Climate worry severity	0,027	0,056	0,470	0,637	-0,084	0,137
Climate worry frequency	0,385	0,056	6,930	0,000	0,276	0,494
_cons	-0,154	0,066	-2,340	0,019	-0,284	-0,025
PEB_total						
WSAS_total	0,049	0,019	2,540	0,011	0,011	0,088
Climate worry severity	0,337	0,038	8,860	0,000	0,262	0,411
Climate worry frequency	0,448	0,038	11,680	0,000	0,373	0,523
_cons	0,603	0,054	11,090	0,000	0,496	0,709
mean(Climate worry severity)	2,358	0,056	42,380	0,000	2,249	2,467
mean(Climate worry frequency)	2,229	0,053	41,730	0,000	2,125	2,334
var(e.WSAS_total)	0,833	0,019			0,795	0,872
var(e.PEB_total)	0,385	0,017			0,353	0,421
var(Climate worry severity)	1,000	,			,	,
var(Climate worry frequency)	1,000	,			,	,
cov(Climate worry severity,Climate worry frequency)	0,887	0,006	144,860	0,000	0,875	0,899

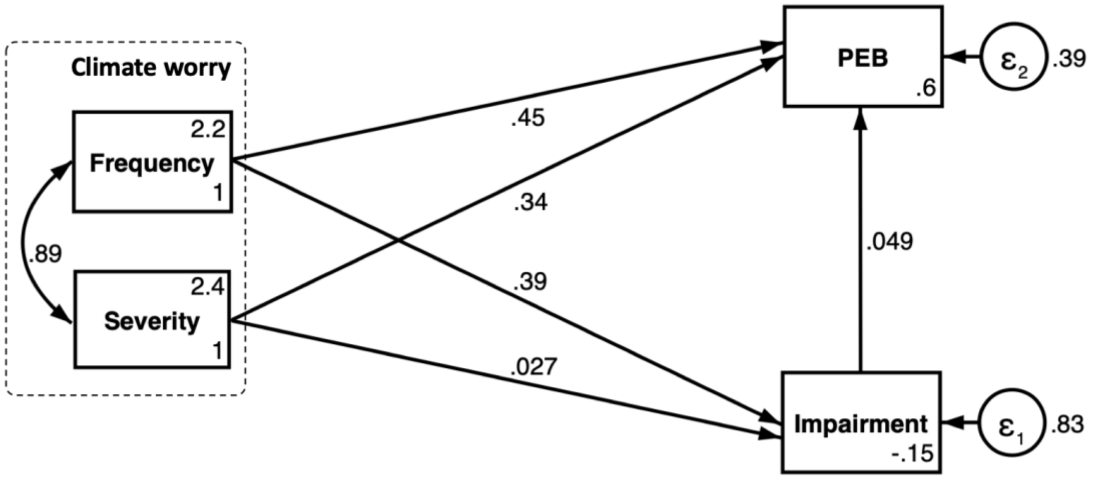

Figure S2: Saturated Structural Equation Model

Final SEM results

Table S3: SEM estimates for the final model

		OIM				
Standardized	Coef.	Std. Err.	z	P>z	[95% Conf.	Interval]
Structural						
PEB						
WSAS_total	0,050	0,019	2,540	0,011	0,011	0,088
Climate worry frequency	0,448	0,038	11,680	0,000	0,373	0,523
Climate worry severity	0,337	0,038	8,860	0,000	0,262	0,411
_cons	0,603	0,054	11,090	0,000	0,496	0,709
WSAS_total						
Climate worry frequency	0,409	0,024	17,150	0,000	0,362	0,455
_cons	-0,144	0,062	-2,310	0,021	-0,267	-0,022
mean(Climate worry frequency)	2,229	0,053	41,730	0,000	2,125	2,334
mean(Climate worry severity)	2,358	0,056	42,380	0,000	2,249	2,467
var(e.PEB)	0,385	0,017			0,353	0,421
var(e.WSAS_total)	0,833	0,019			0,796	0,872
var(Climate worry frequency)	1,000	,			,	,
var(Climate worry severity)	1,000	,			,	,
cov(Climate worry frequency,Climate worry severity)	0,887	0,006	144,860	0,000	0,875	0,899
